# From “the Moon Is Rounder Abroad” to “Bravo, My Country”: How China Misperceives the World

**DOI:** 10.1007/s12116-021-09322-z

**Published:** 2021-02-15

**Authors:** Haifeng Huang

**Affiliations:** Department of Political Science, University of California, Merced, CA USA

**Keywords:** Misinformation, Xenophilia, Self-aggrandizement, Propaganda, Backlash

## Abstract

For a long time, since China’s opening to the outside world in the late 1970s, admiration for foreign socioeconomic prosperity and quality of life characterized much of the Chinese society, which contributed to dissatisfaction with the country’s development and government and a large-scale exodus of students and emigrants to foreign countries. More recently, however, overestimating China’s standing and popularity in the world has become a more conspicuous feature of Chinese public opinion and the social backdrop of the country’s overreach in global affairs in the last few years. This essay discusses the effects of these misperceptions about the world, their potential sources, and the outcomes of correcting misperceptions. It concludes that while the world should get China right and not misinterpret China’s intentions and actions, China should also get the world right and have a more balanced understanding of its relationship with the world.

Chinese people have always had only two terms of address for foreigners: either “beasts” or “majesties.” They have never been called friends, nor said to be the same as us.

— Lu Xun (1919), “Random Reflections No. 48”

## Introduction

How does the Chinese public perceive the world? Studies on Chinese citizens’ international outlooks focus on subjective attitudes toward foreign countries and international affairs including nationalistic sentiments (Gries and Sanders 2016; Han and Zweig 2010; Johnston 2004; Shi et al. 2011; Woods and Dickson 2017; Zhang et al. 2018).^1^ Some scholars and analysts have also examined the Chinese state’s perspectives on the world in terms of international relations (Feng et al 2019; Fu 2016; McMaster 2020; Nathan and Scobell 2012; Shambaugh 1993). In contrast, people’s factual information about the world, including their perceptions of foreign socioeconomic prosperity and China’s global image and standing, has received far less attention. In short, we do not yet have sufficient understanding of what the Chinese society thinks the world is like.

This situation is not unique to China. The large literature in political science on citizen knowledge has focused on domestic knowledge in democratic countries (e.g., Delli Carpini and Keeter 1996; Lupia and McCubbins 1998; Prior 2007; Zaller 1992), generally ignoring how knowledge about the world can also have significant influence on people’s political attitudes. Recently, however, an emerging literature on international bench-marking has shown that information and perceptions about foreign countries’ performances or domestic-foreign comparisons have significant influences on individuals’ political attitudes (Aytac 2020; Huang 2015; Kayser and Peress 2012). In line with this literature, this essay argues that, besides subjective values and attitudes, factual information about the world and China’s position in it also shapes how the Chinese public views China and its international relations.

I will discuss in particular how the Chinese society often does not have a proper and balanced understanding of foreign nations, which negatively affects China’s own development and foreign relations. The Chinese public tends to have either an overly romantic view of the outside world, thinking “the moon is rounder abroad” (月亮是外国的圆), or an overly bleak view of foreign countries, overestimating China’s power, popularity, and influence in the world, which is perhaps best illustrated by the title of an influential 2018 documentary film, *Bravo, My Country* (厉害了, 我的国). The former view has characterized much of the Chinese society since China’s opening to the outside world in the late 1970s, and the latter has become more conspicuous in recent years. Both (mis)perceptions are unhealthy, with the former sometimes leading to an inferiority complex and helping engender a large-scale exodus of students and emigrants to foreign countries, while the latter has resulted in arrogance and egotism and contributed to China’s overreach on the global stage in recent times.

As has been shown in previous studies, information is essential for opinion formation and decision making, and having wrong information can be more detrimental than having no information (Gilens 2001; Kuklinski et al. 2000). Similar to the literature on citizen knowledge, these studies, as well as the increasingly large literature on fake news and rumor (Berinsky 2017; Nyhan et al. 2017; Huang 2017a), also focus on domestic issues and events. If ignorance and misinformation about one’s own country can be pervasive, as prior research has shown, misperceptions about foreign nations will likely be even more serious. This essay thus reviews existing studies and evidence, both qualitative and quantitative, to discuss the effects of citizens’ misinformation and misperceptions about the outside world, their potential sources, and the outcomes of information correction.

Facing a China ascending on the world stage, often with clumsily aggressive behavior (Hille 2020), much of the world has become alarmed about China’s potential ambitions and intentions in recent years (Diamond 2019; Schell and Shirk 2019). The risk of misinterpretation and overreaction is enormous, and scholars have rightly called for getting China right (Fravel et al. 2021; Weiss 2019). What has been less emphasized is that China should also get the world right, not only at the government level but also at the societal level, since both unwarranted self-loathing and overestimation of one’s position in the world can have grave consequences.

## The Moon Is Rounder Abroad: The Inferiority Complex

### Overestimating Foreign Prosperity and Its Consequences

As a poor and backward China started to open to the world in the late 1970s and early 1980s, fascination and enchantment with the West swept the country and gave rise to the popularity of the colloquial expression “the moon is rounder abroad.”^2^ Some observers even labeled young Chinese people’s naive attitudes toward foreign countries and their uncritical acceptance of anything Western as “reverse racism” (see Rosen 2010). Admiration of Western countries in the beginning decade of the reform era was all-around, covering culture and philosophy, political institutions, and material wealth, as reflected in the 1988 hit TV documentary *River Elegy* (De Jong 1989).^3^ After the Tiananmen Movement failed in 1989, the Chinese public’s imaginings of the West focused more narrowly on socioeconomic conditions, perhaps in line with the Chinese society’s almost single-minded pursuit of material advancement since the 1990s.^4^

The perceptions about the rounder moon abroad are often characterized by significant over estimation of Western prosperity and purity. For example, Vanessa Fong’s (2011) ethnographic account of a large group of Chinese students going abroad for college in the 2000s details how they grew up admiring the images of the developed world, despising China’s problems, and yearning for lifestyles and opportunities available abroad. She bluntly observes that “[m]any Chinese citizens referred to developed countries as paradise (*tiantang*)” (p. 51), and that “just about everyone I met complained incessantly about China’s inferiority to developed countries” (p. 54). When the realities abroad did not quite meet their high expectations, they “increasingly saw China as a paradise lost and the developed country they once imagined as paradise as a barren, unsatisfying trap” (p. 200),^5^ even though they also developed appreciation for some freedoms they could enjoy abroad but not in China. Other qualitative studies of Chinese overseas students have similarly found that they usually had rose-colored expectations before they went abroad (Hansen 2015; Kajanus 2015; Gamst Page 2019). Journalists have found similar stories with Chinese immigrants to the US and Europe (Guo 2017; Rong 2017).

This phenomenon is not China-specific. Studies have suggested that immigrants to Western countries from other nations often have similar erroneous information or expectations about opportunities in their destinations (Borjas and Bratsberg 1996; Stillman et al. 2015). There are also reports of African immigrants disillusioned by China (Kuo 2016). But the phenomenon is perhaps more consequential for China as one of the largest origin countries of international students and migrants (Royce 2019; Shah 2015): For example, currently about one-third of all international students in the US hail from China, more than the next seven countries combined. And unrealistic expectations do not just affect people studying or moving abroad. The phenomenon of worshiping things foreign has been evident in all corners of China. Chinese firms like to give their products foreign-sounding brand names in order to attract customers (Levin 2014), and cities across the country have built numerous replicas of American and European architecture and sometimes entire foreign towns (Bosker 2013). Sometimes Chinese internet users would even attribute the quality of a mostly domestically built city drain system to the work of foreign engineers over a century ago (Neidhart 2015).

The issue also has larger political consequences in addition to affecting the personal well-being of transnational migrants and students. With a series of surveys and survey experiments from 2011 to 2014, I have found that overestimation of foreign socioeconomic prosperity (e.g., per capita income, life expectancy, and public safety) leads to lower evaluations of China, more dissatisfaction with the Chinese government, and higher inclinations to exit the country among the Chinese public, while underestimation has no clear effects (Huang 2015, 2017b). Correcting misinformation about foreign socioeconomic conditions often improves individuals’ domestic attitudes and reduces their inclination to move abroad. The effect of international political knowledge, on the other hand, is not significant or consistent, suggesting that Chinese citizens’ aspirations for foreign countries is more socioeconomic than political in nature.^6^

**Fig. 1:**
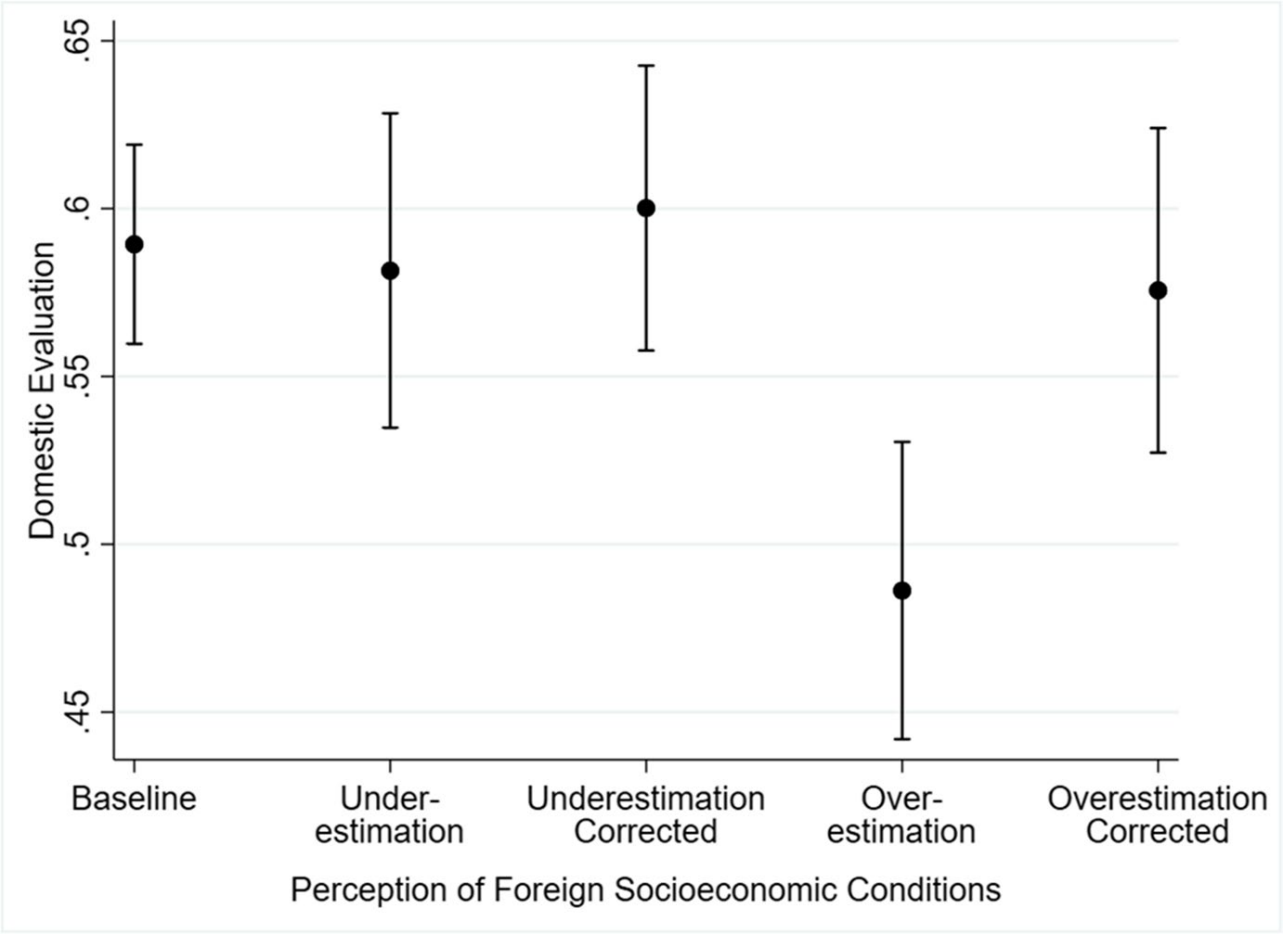
How misperceptions and corrections about foreign socioeconomic prosperity affect domestic evaluation. *Note*: Predicted values with 95% confidence intervals. Domestic evaluation is aggregated from evaluations of China’s current overall situation, future prospects, and political system, and is rescaled to lie between 0 and 1. Huang (2015) analyzes a different set of outcome variables

Figure 1, produced using data from a 2012 online survey experiment referenced in Huang (2015),^7^ illustrates how misperceptions and corrections about foreign socioeconomic conditions affect the Chinese public’s domestic attitudes. In the experiment, conducted with a demographically diverse group of Chinese internet users recruited from a crowd-sourcing platform, the respondents were first asked a set of factual questions about socioeconomic conditions in the Organization for Economic Cooperation and Development (OECD) countries, particularly the United States. They were then randomly assigned to the control and correction conditions. Respondents in the correction condition were informed of which questions they answered wrong and of the correct answers to those questions according to official statistics from the US government and United Nations, while those in the control condition did not receive such information. All respondents were then asked a series of outcome questions, including how satisfied they were with China’s overall current situation, how optimistic they were about China’s future prospects, and how appropriate they thought China’s political system was. These three variables are aggregated into the *Domestic Evaluation* variable, rescaled to lie between 0 and 1 for easy interpretation, in Fig. 1. Figure 1 also groups the respondents into categories according to whether they systematically overestimated or underestimated socioeconomic prosperity in OECD countries, and whether they received corrections. The baseline group refers to respondents in the control condition who had roughly balanced estimates of foreign socioeconomic conditions.

As shown in Fig. 1, the overall domestic evaluations of the first three groups (the baseline group, the group that underestimated foreign socioeconomic prosperity, and the group that underestimated foreign prosperity but then received corrections) were similar. In other words, underestimating foreign socioeconomic prosperity did not significantly affect the respondents’ domestic attitudes. The group that overestimated foreign prosperity, however, had a significantly lower level of domestic evaluation than the first three groups. On the other hand, the group of respondents who overestimated foreign prosperity but then received corrections had similar levels of domestic evaluation as the first three groups, indicating that correcting the overestimating respondents’ misinformation returned their domestic evaluation to a more “normal” level. The magnitude of change on domestic evaluation due to information correction was about nine percentage points. Clearly, overestimation of foreign socioeconomic prosperity made people more critical of China and the Chinese government, while correcting misperceptions improved their domestic attitudes.

### Sources of Misperceptions about Foreign Prosperity

A critical question is why misperceptions about foreign countries are so prevalent in China after decades of opening to the world. Part of the reason is likely that most Chinese people have little direct experience living in foreign countries so that their perceptions about the outside world are based on second-hand information from interpersonal communication, media, and the internet (Dong et al. 2013; Fong 2011). In her participant observatory study, for example, Fong (2011) discovers that Chinese people returning from abroad tend to talk only about positive aspects of their experiences abroad and to downplay unpleasant aspects or redefine their original goals for going abroad, perhaps to console themselves or save face in front of others.

As for the media, any acute observer of the Chinese internet just a few years ago would have had no problem seeing that it is full of popular but misleading tales that overly romanticize foreign countries (Darnton 2012; Neidhart 2015; Yung 2011). An analysis of a rich dataset of social media posts published in 2013 on Weibo, China’s counterpart to Twitter, shows that the predominant narrative about the United States among public opinion leaders is pro-US, and nationalist sentiment may arise as the pro-US narrative is called into question by more information (Zhang et al. 2018). A common fascination of the Chinese public is with foreign countries’ high income levels, supposedly low living costs, and attractive welfare benefits. For example, an influential national television host once famously (and erroneously) remarked: “What is wrong with our telephone charges? What on earth should be charged, and what should not? In Los Angeles, USA, cell phone plans are only 9.9 dollars for the whole year. Can you believe it?” (Huang 2015, p. 616, footnote 4). For another example, Weibo once had a spate of bogus but well-followed accounts from self-proclaimed foreign “immigration bureaus” that provided inaccurate and misleading descriptions about the countries they were supposed to represent. The “Indian Immigration Bureau,” for instance, claimed that India offers its people not only subsidized but free treatment in its luxurious hospitals, in addition to free and unlimited rides on most trains (Huang and Yeh 2019, p. 618). The low living cost part of the fascination is particularly surprising, given the well-known fact that China’s gross domestic product is significantly larger when calculated with the purchasing power parity than with the market exchange rate.^8^

The consequences of this kind of misinformation are well illustrated by the story of a cyclist couple self-nicknamed “Gulu Sisi,” once well publicized on their own Weibo account.^9^ In 2015 the couple launched a global cycling tour with only a modest amount of savings, thinking that “except for airfare, basically everything else is a lot cheaper abroad than in China” (Huang 2017b, p. 194). Starting the tour in the US, however, they realized that “the China-US price comparison we have seen earlier is misleading. The US is actually quite expensive. Going to the supermarket by bus alone cost us 100 RMB yuan” (p. 194). Consequently, they had to save on basic food items and sometimes even slept on the street to reduce travel costs, until a few days later they were sadly hit by an uninsured motorist, which left them severely injured and facing a medical bill of about 600,000 US dollars. Fortunately, they were able to return to China to continue treatment thanks to the help of local volunteers and a Chinese health insurance company.^10^

How would exposure to more accurate and reliable information sources about foreign countries, such as foreign media, influence people’s political attitudes? A methodological challenge here is obviously that information exposure is self-selected, and we must separate the effect of information on attitudes from the political preferences that lead to the information exposure in the first place. To address this issue, Huang and Yeh (2019) designed a procedure called “randomized realization of self-selected treatments” (RRSST), which combines voluntary selection with randomization to reveal the effect of media exposure, after controlling for self-selection of media exposure. Following the RRSST design, study participants are first asked to select media content that they would like to consume. Then, among individuals who selected the same content, some are randomly chosen and given the content to read, while others are not. By comparing the post-treatment attitudes of the individuals who have read the media content and those who have the same exposure preference but do not actually read the content, we can see how information from media affects the opinions of individuals interested in accessing such information. This simple procedure can be regarded as a modification of the standard patient preference trial design recently introduced into political science and media studies (Arceneaux and Johnson 2013; Gaines and Kuklinski 2011; Levendusky 2013), but is potentially more straightforward than the standard design.

With this design, Huang and Yeh (2019) find that Chinese citizens with higher pro-Western orientation and lower domestic evaluations are more inclined to read foreign media content that is positive about foreign socioeconomic performances or negative about China. Perhaps more importantly, reading foreign media often improves rather than worsens the domestic evaluations of Chinese citizens who self-select such content. This is apparently because mainstream Western media reports of their own country’s socioeconomic situations are generally more accurate and realistic than the overly rosy information about foreign countries that popularly circulates in China. Reading foreign media content thus serves as a correction to initial imaginings.

The results from the aforementioned studies are counterintuitive. Conventional wisdom assumes that, due to media censorship and government propaganda, citizens in authoritarian countries lack sufficient information about the performance and prosperity of advanced democracies, and that more information about the outside world will inspire them to demand changes at home. These studies suggest that the opposite is often the case: In a rapidly developing country, a sizable population may have views of foreign countries that are too rosy rather than too bleak; more accurate and ample information may improve rather than undermine domestic stability. Censorship of foreign information, in this sense, is counterproductive.

### The Moon Is Not Rounder Abroad

Several of the aforementioned studies have shown that correcting overestimation of foreign socioeconomic prosperity can improve a person’s evaluation of domestic circumstances. The consequence of realizing that the moon is not necessarily rounder abroad is also reflected in a seemingly puzzling phenomenon among Chinese overseas students. While some Chinese students have developed a deep appreciation of Western societies after embarking on overseas study, many others not only do not become more pro-democracy, but actually become more nationalistic (Chen and Chin 2017; Fish 2018; Tea Leaf Nation 2015). The cause may be complicated and multifaceted, but part of the reason is that the students’ initial rosy image of their host countries was sullied after arrival.^11^ Two recent campus-wide surveys of Chinese students and scholars at Purdue University show that those whose views of the US worsened after coming to the US outnumber those whose opinion improved, whereas the reverse is true for views on China (Purdue CRCS 2016, 2018). A smaller survey among Chinese students in Germany similarly found that most students’ views on China became more positive after arriving in Germany (Mao 2020). Scholars who hope that overseas study and cultural diplomacy will contribute to China’s democratization may thus be disappointed, at least in the short run. To some extent, this is a natural outcome of adverse selection: People who lack a proper appreciation of advanced democracies are the ones who need to learn from the West the most, but the people most likely to pay the financial, emotional, and other costs of going abroad are those who already admire the West very much, and they can only be disenchanted. This is the winner’s curse as applied to transnational study and migration.

Another, perhaps ironic, consequence of the prevalent sense of inferiority is that it has contributed to a political and social backlash against openness and internationalism in China, just as the cultural backlash against societal changes in the US and UK may have led to the rise of Donald Trump, Brexit, and “Make America Great Again” (Norris and Inglehart 2019). Chinese media now regularly publish articles denouncing worship of things foreign or “US sycophants.”^12^ At the highest level of power, the state also responded with the Four Self-Confidences doctrine (“confidence in our chosen path, confidence in our political system, confidence in our guiding theories, and confidence in our culture”), which helped push the pendulum to the other extreme, as analyzed in the next section.

## Bravo, My Country: The Backlash

In the last few years, it appears that there have been some significant changes in public sentiments in China with regard to the country’s relationship with the world. The most conspicuous public perception regarding China and the world on the internet and social media is no longer overestimating foreign prosperity and performance but overestimating China’s relative power, wealth, and global influence. Complacency and swagger about China’s rise and “breakthrough” pervade the society and enjoy wide public appeal (Buckley 2020). While overestimating the achievement of ancient Chinese civilization has long been commonplace (Huang and Liu 2019), overestimating China’s current standing in the world is a relatively new phenomenon. As a country becomes richer and more prosperous, underestimation of foreign countries’ positions may naturally arise and matter more than overestimation of foreign countries (Huang 2015). This is shown most clearly with the coming of age of a new generation that grew up in stability and relative prosperity. But pervasive official propaganda about China’s rise in the world, and societal backlash against the inferiority complex, which drives the popularity of social media narratives exaggerating China’s power and the respect it commands in the world, have also been critical in the process.

### A New Generation

Part of the reason for the perceptual and sentimental change is related to demographic shifts, especially the coming of age of the so-called “post-1990” generation (in particular, the post-1995s), or China’s Generation Z. This is a generation born during China’s golden era of rapid growth and overall stability. It is a generation growing up with the Olympics, high speed rails, and mobile payments, but with few direct personal memories of poverty or turmoil. Naturally, these experiences in people’s formative years would shape their memory and political attitudes (Jennings and Zhang 2005). Thus, while the somewhat earlier, post-1980 generation of “angry youths” have diverse opinions ranging from nationalistic to China-critical (Osnos 2008; Yang and Zheng 2012), many of the post-1995s, often dubbed “little pinks,” have a high level of patriotism and national pride (Fang and Repnikova 2018).

For many people in this generation, the star of the West is dimming, even if Western culture has become part of their life (Hornby 2019; Qin 2019). Easy access to money and luxury makes many of them blindly optimistic, overestimating China’s prosperity and power while unaware of the country’s inequality and potential fragility hidden behind some shining urban skylines. In a recent clash between mainland Chinese students and pro-Hong Kong protesters in Canada, for example, the former derided Hong Kongers as paupers,^13^ notwithstanding the fact that Hong Kong’s per capita income is still significantly above that in the mainland. There is some indication that the trade and technological war between the US and China has given many people in China a reality check and tempered their sense of superiority. On the other hand, America’s fumbling response to the Covid-19 pandemic has enhanced their doubt about the US (Wertime 2020). Either way, it is apparent that the new generation sees China quite differently than did earlier generations.

While some of the increased self-confidence of the young generation is a justified and healthy response to China’s rise and the self-loathing among some in the earlier generations, too often it also fuels excessive grassroots nationalism. In recent years China’s youths have engaged in online activism of various sorts, such as the famed “Diba Expedition” in 2016, in which they flooded Taiwanese leader Tsai Ing-wen’s Facebook page with patriotic and pro-unification comments following the independence-leaning Tsai’s electoral victory (Liu 2019). Young artists like Wuhe Qilin have also been at the forefront of fanning nationalism, even directly influencing China’s foreign relations, such as the recent tension with Australia (Buckley 2020; Feng 2020). Many of the youths are sometimes even intolerant of moderate criticism of China, as shown in their vehement denunciations of the Wuhan-based writer Fang Fang for her diary chronicling the city’s experience during the coronarivus lockdown (Lau and Xie 2020). They accused Fang of “giving a knife” for foreign nations to attack China, apparently unaware that it is their own behavior that has made China look worse. This lack of understanding of the wider world can also lead to embarrassing debacles when they debate foreign internet users on international platforms, as they recently did over a few very minor comments on Hong Kong, Taiwan, and Covid-19 by two Thai celebrities (Griffiths 2020; Reuters 2020a). Even some Chinese diplomats of the relatively younger and tech savvy generation have broken with tradition and are now acting aggressively, feuding with foreign countries like “wolf warriors,” including openly backing conspiracy theories about the origin of the Covid-19 virus on social media, and worsening China’s international image in the process (Hille 2020; Shepherd 2020).

### Propaganda and Clickbait

The latest trend in Chinese public sentiments is also a backlash to the prevailing inferiority complex of earlier times. Part of the backlash is natural societal reactions, but perhaps a more important source is relentless government policies that clamp down on discordant voices and excessive efforts to promote national self-confidence, embodied in the Four Self-Confidences doctrine mentioned earlier. As a result, self-aggrandizement instead of moderation or self-criticism become the “main melody” in the media. While China has traditionally insisted that it is a developing country, in the last decade or so the buzzword in official media is instead “great power,” as in “the rise of a great power,” “the diplomacy of a great power,” and even “a great power’s battle with the epidemic.”^14^ The documentary film *Bravo, My Country*,^15^ released by the state broadcaster China Central Television in 2018, perhaps best captures the self-aggrandizement. The documentary touted China’s recent achievements in areas including science and technology, infrastructure, and military modernization. Aided by its stunning visuals and sometimes compulsory screenings, the movie quickly became China’s top-grossing documentary of all time and received high ratings domestically (Huang 2018; Zheng 2018). For another example, during the coronavirus crisis China’s propaganda apparatus has blanketed the nation with features about the country’s effectiveness in controlling the pandemic and its provision of aid to other countries, highlighting other countries’ inadequate responses and suppressing discussions about China’s own mistakes, all of which have fueled the public’s scorn of foreign countries, such as the common lament that they cannot even “copy homework” from China in dealing with the virus (Huang 2020a; Li 2020; The Economist 2020).

In tandem with government propaganda, promoting nationalism has also become good business, as reflected in the commercial success of hyper-nationalistic military action movies like *Wolf Worrier 2 *and *Operation Red Sea* (The Economist 2017). The former movie, released in 2017, still holds the title of the highest-grossing movie ever in China. Movies like these and *Bravo, My Country* may well have given many people exaggerated pride in China’s supposed formidable power and advanced technology. Sentiments reflected in sayings like “Anyone who affronts my China will pay, even if they are far away,” adapted from an ancient classic and made popular by *Wolf Warrior 2*, likely also underlie the Chinese public’s firestorm over a rather insignificant and quickly deleted tweet by an American basketball team manager supporting Hong Kong’s protest movement in 2019 (Deb and Stein 2019). The public outcry also prompted Chinese state media and commercial entities with international ties to overreact and thus turned a minor incident into a major fiasco harming China’s global image (Wang 2020).

For-profit online publications that seek to monetize views and shares have also taken a hint from this trend, and now often play up inaccurate but sensational (sometimes entirely made-up) stories about foreign countries’ plight and problems. During the Covid-19 pandemic, for example, Chinese social media was awash with copy-paste stories that exaggerated hardship and chaos in various foreign countries that differ from each other only in the country’s name and a few minor details (Zhang 2020). This kind of content not only targets China’s domestic audience but also Chinese nationals living overseas, as in the case of *College Daily*, which is popular among some Chinese students abroad (Zhang 2019).

Chinese social media have also been full of nationalist-chauvinist fake news exaggerating China’s influence and attraction on the world stage. Recently, for example, a spate of WeChat posts claim that various countries or territories want to “return to Chinese sovereignty,” which prompted Kazakhstan, one of the countries implicated, to summon the Chinese ambassador to protest (Reuters 2020b). Consequently, WeChat deleted over one hundred accounts disseminating such fake news, and China’s official media also criticized these social media accounts as being “sick” (People’s Daily 2020).

With such a misinformation bubble, as well as the prohibition of “frivolous comments on the Center” and loyalty-signaling local officials’ competitive reinforcement of central policies, a spiral of silence (Noelle-Neumann 1993) easily develops, and opinion leaders, policy advisors, and ordinary people alike withhold their dissenting opinions. The result is that, while it used to be common for public intellectuals and social media influencers to admire the purity of foreign countries and be critical of China’s ills, with the silencing of many so-called “Big-Vs” (verified accounts with large followings) it is now more prudent for celebrities and others to signal patriotism during internet incidents. While a few years ago viral social media posts about China-foreign comparisons tended to exaggerate foreign prosperity and virtues, it is now much easier to see posts exaggerating China’s power, technology, and global influence, or foreign instability and chaos, being circulated far and wide. This is the social backdrop of China’s underestimation of foreign strength and overestimation of its own capability, leading to the abandonment of Deng Xiaoping’s foreign policy dictum of “keeping a low profile and biding time.”

### Inconvenient Information

The above discussion suggests it is important to examine the extent to which the Chinese public may overestimate China’s global standing and the potential effects of correcting misperceptions. I therefore conducted an online survey experiment in March 2020 with over 2000 respondents recruited through a market survey company, whose demographic profiles largely resemble China’s general internet population. While overestimation of a country’s world position can include its power, prosperity, and popularity, this survey focused on the last item: how the Chinese public thinks about China’s popularity and image in the world.

The survey first asked respondents six questions on China’s global image, including the median percentage of people in different parts of the world with positive views of China and the share of Hong Kong residents with a favorable view of the mainland Chinese government (for details, see Huang 2020b). Percentages ranging from low to high were provided as choices for each question, and the respondents’ answers can be compared to results from benchmark surveys by major global and local polling organizations to see if they overestimated or underestimated China’s global popularity.

Results show that the respondents overwhelmingly overestimated the positiveness of China’s global image. For example, while the 2019 global attitudes survey by the Pew Research Center shows that a median of 35 percent of people in North American and Western European countries had a positive view of China, over 83 percent of the respondents chose answers that overestimated the extent to which China’s image was positive in these countries, with about 54 percent thinking that the median was above 60 percent. Similarly, though a 2020 poll conducted by the Hong Kong Public Opinion Research Institute indicates that only 22.6 percent of Hong Kong residents had positive opinions about the mainland Chinese government, 69 percent of my respondents thought the majority of Hong Kong residents had positive views on the mainland government. Answers to other questions are similar. In fact, over 61 percent of the respondents overestimated China’s popularity in all six questions.

The survey also had an experimental stage to assess the effects of correcting misperceptions. Specifically, in this stage a group of randomly assigned respondents was given results about the above questions from global benchmark opinion polls, for questions where their answers were different from the benchmark polls, while those in the control condition were not given the information. Then all respondents were asked a series of questions measuring their evaluation of China, its governing system, and its prospects of success in the world. To be consistent with the previous section, here I focus on the outcome questions on China’s overall situation, China’s future prospects, and China’s political system, which will again be aggregated into the variable *domestic evaluation*.

**Fig. 2:**
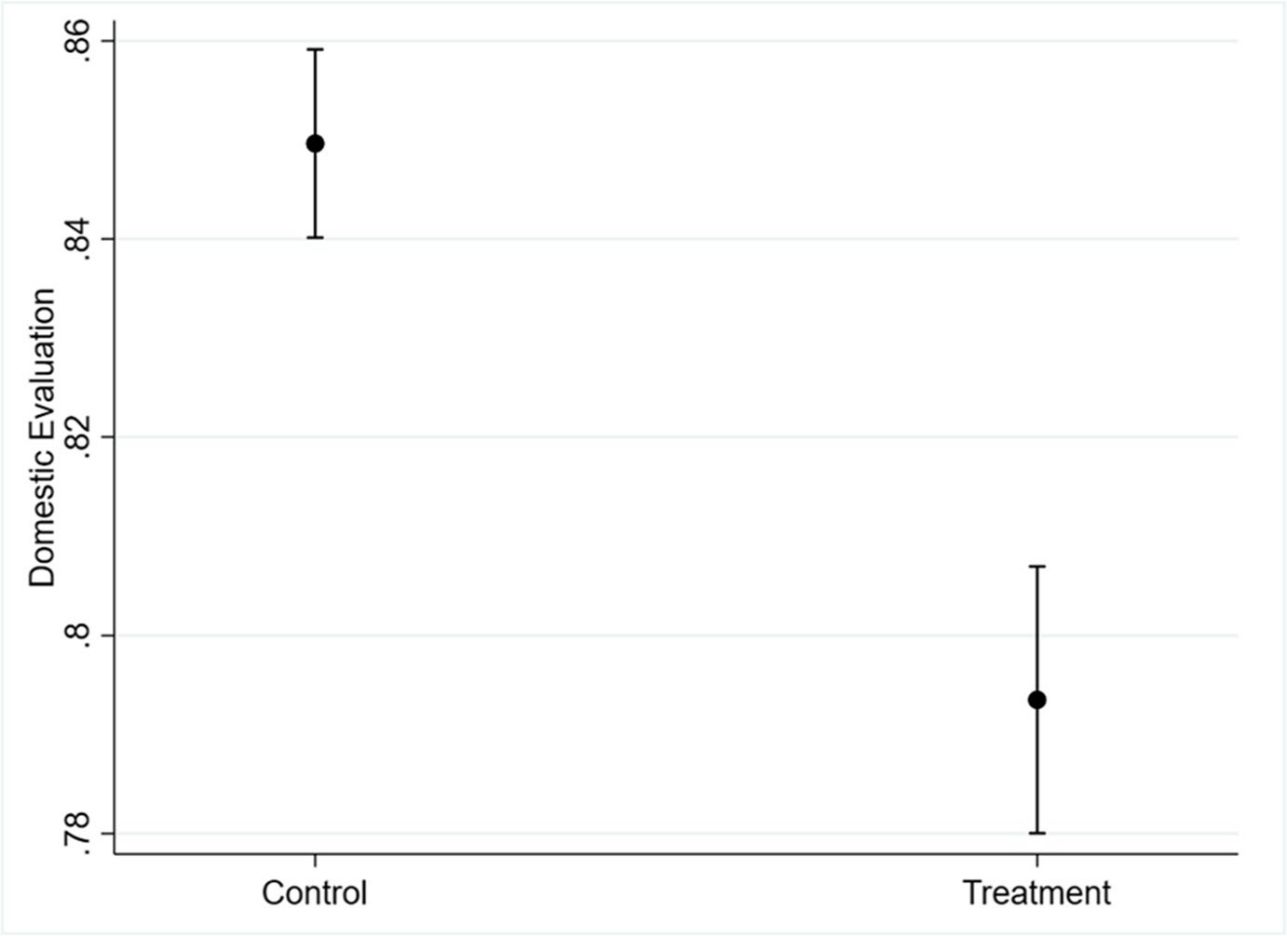
Effect of correcting misperceptions about China’s global image. *Note*: Predicted values with 95% confidence intervals. Domestic evaluation is aggregated from evaluations of China’s current overall situation, future prospects, and political system, and is re-scaled to lie between 0 and 1. Huang (2020b) conducts a different set of analyses

Figure 2 shows the effect of correcting misinformation about China’s global image: Receiving information about how China is actually viewed around the world significantly reduced the respondents’ evaluation of China. The magnitude of change is 5.6 percentage points. Note that some respondents also underestimated the positiveness of China’s global image in some of the questions, but since the number of overestimating answers far exceeded those of underestimating answers, the effect of information intervention was driven by correcting overestimation of China’s global popularity.

The survey thus shows that being immersed in a one-sided information bubble about the glory of a rising power has made the Chinese public overly complacent and led it to significantly overestimate the country’s global standing. At the same time, the experiment indicates that people are responsive to new information, which moderates their evaluation of China, rather than resisting updates through motivated reasoning. It also suggests that propaganda may eventually backfire by excessively raising expectations to levels that cannot be met, which then leads to letdowns once people are exposed to more accurate information.

## Discussion and Conclusion

The swinging of Chinese society from “the moon is rounder abroad” to “bravo, my country” proves the observation of Lu Xun, a leading figure of modern Chinese literature and a penetrating social critic, quoted at the beginning of this essay. Since China first opened its doors to the outside world in the late 1970s, the Chinese public’s perception of the world and China’s relationship with it can be characterized by romantic imagination and admiration of the West, particularly its socioeconomic performance and prosperity. These perceptions and sometimes overestimation of Western prosperity increased Chinese people’s affinity for the West, reduced the Chinese public’s satisfaction with China’s domestic development and governing system, and helped make China one of the largest sending countries of international students and immigrants.

More recently, a large section of the Chinese public has displayed a rather different tendency in their perceptions about China’s relationship with the rest of the world: They significantly overestimate China’s power, influence, and popularity on the global stage. This is partly due to China’s economic and political rise, including the emergence of a new generation born in relative affluence, but also to the relentless government and private-sector propaganda about China as a great power in the world. While overestimating foreign socioeconomic prosperity and overestimating China’s influence and popularity are not necessarily contradictory, the new trend reflects a new kind of sentiment and worldview. Such complacency and self-aggrandizement give many people a false sense of national superiority and may have bolstered China’s wolf-warrior style of assertive and even jingoistic diplomacy, unnecessarily intensifying conflicts with many countries and eventually harming China’s own interests.

One may hope that these issues arise because China is a relative newcomer in engaging with the world. When a formerly closed society opens itself, people start to acquire some limited information about the outside world. But a little knowledge can be a dangerous thing, as it can lead to either unwarranted self-loathing or complacency. The fact that the Chinese society has not fundamentally changed since Lu Xun’s observation over a century ago, however, suggests that the process of becoming a normal country that treats foreign nations as equals and “the same as us,” rather than looking up to them with worship or down on them with disdain, will be a long and arduous one.

Finally, the current research has theoretical implications for the study of citizen knowledge and opinion formation. As mentioned earlier, research on citizen knowledge and misinformation has focused on domestic issues. In contrast, the research reviewed here shows that knowledge and (mis)perceptions about foreign countries, whether about what those countries are like or how people in those countries think of one’s own country, also have critical importance for people’s attitudes and, consequently, behavior. In the interconnected world, opinion formation is an international process, not one confined to the domestic arena.

## Footnotes

1 Conversely, there is a small literature on the attitudes of citizens of other countries toward China (Aldrich et al. 2015; Gries et al. 2011; Li 2021; Page and Xie 2010).

2 This expression is usually made in the context of criticizing people for blindly worshiping foreign countries.

3 Some cultural and political idealization of the US continues to this day, especially among intellectuals (Lin 2020b).

4 Even during the Tiananmen Movement, material possessions and Western lifestyle were a big part of the students’ pursuits. As the student leader Wu’er Kaixi put it in an interview (Gordon and Hinton 1996): “So what do we want? Nike shoes. Lots of free time to take our girlfriends to a bar. The freedom to discuss an issue with someone. And to get a little respect from society.”

5 One student in her study observed: “Probably 90 percent of Chinese students abroad think life abroad is not as good as they thought it would be” (p. 194).

6 Awareness of political instability in other countries can even increase satisfaction with China (Huang 2015).

7 Huang (2015) mainly uses data from a 2011 survey and a 2014 survey experiment, with similar findings.

8 One likely reason is the soaring housing prices in many of China’s urban areas in recent times, which can be significantly higher than prices in many parts of advanced countries.

9 Their Weibo account is no longer available.

10 See https://bit.ly/3mNkE50 (last accessed October 26, 2019).

11 The disappointment is, of course, not restricted to socioeconomic prosperity but can also be about culture and education (Lin 2020a).

12 See, e.g., China Central Television, “A small number of people’s American worship mentality is a disease.” https://bit.ly/3mLFBgE (last accessed December 16, 2020).

13 See https://bit.ly/2Jijvoa (last accessed May 8, 2020).

14 The last expression is the title of a book purportedly showing the extraordinary success of China in fighting the coronavirus, the skill of the Chinese leadership, and the superiority of the Chinese political system. See https://bit.ly/2WLSWL6.

15 The movie’s official English title is *Amazing China*. *Bravo, My Country* is a more literal translation of its Chinese title. See Fang (2018) for a discussion.
